# Reliability of Self-Reported Mobile Phone Ownership in Rural North-Central Nigeria: Cross-Sectional Study

**DOI:** 10.2196/mhealth.8760

**Published:** 2018-03-01

**Authors:** William Nii Ayitey Menson, John Olajide Olawepo, Tamara Bruno, Semiu Olatunde Gbadamosi, Nannim Fazing Nalda, Victor Anyebe, Amaka Ogidi, Chima Onoka, John Okpanachi Oko, Echezona Edozie Ezeanolue

**Affiliations:** ^1^ Global Health Initiative School of Community Health Sciences University of Nevada, Las Vegas Las Vegas, NV United States; ^2^ Caritas Nigeria Abuja Nigeria; ^3^ Global Health Initiative School of Community Health Sciences University of Nevada Las Vegas Las Vegas, NV United States; ^4^ Department of Community Medicine University of Nigeria Enugu Nigeria

**Keywords:** reliability, phone ownership, resource-limited setting, cell phone use, rural population, developing countries, self report, Nigeria, telemedicine

## Abstract

**Background:**

mHealth practitioners seek to leverage the ubiquity of the mobile phone to increase the impact and robustness of their interventions, particularly in resource-limited settings. However, data on the reliability of self-reported mobile phone access is minimal.

**Objective:**

We sought to ascertain the reliability of self-reported ownership of and access to mobile phones among a population of rural dwellers in north-central Nigeria.

**Methods:**

We contacted participants in a community-based HIV testing program by phone to determine actual as opposed to self-reported mobile phone access. A phone script was designed to conduct these calls and descriptive analyses conducted on the findings.

**Results:**

We dialed 349 numbers: 110 (31.5%) were answered by participants who self-reported ownership of the mobile phone; 123 (35.2%) of the phone numbers did not ring at all; 28 (8.0%) rang but were not answered; and 88 (25.2%) were answered by someone other than the participant. We reached a higher proportion of male participants (68/133, 51.1%) than female participants (42/216, 19.4%; *P*<.001).

**Conclusions:**

Self-reported access to mobile phones in rural and low-income areas in north-central Nigeria is higher than actual access. This has implications for mHealth programming, particularly for women’s health. mHealth program implementers and researchers need to be cognizant of the low reliability of self-reported mobile phone access. These observations should therefore affect sample-size calculations and, where possible, alternative means of reaching research participants and program beneficiaries should be established.

## Introduction

More than 443 million of the 6 billion mobile phone subscribers worldwide are in Africa [1]. Additionally, sub–Saharan Africa has experienced the highest rate of growth in mobile subscriptions globally within the last decade [2]. The penetration level of the subscriber identity module, which in 2010 was just approaching 50%, is now forecasted to be close to 100% [2], with expectation of full coverage by 2021. This burgeoning growth is even more visible in West Africa, which as of 2015 had about 40% of all mobile subscriptions in sub–Saharan Africa [3]. Nigeria has the largest mobile phone market in West Africa and, with over 150 million subscribers [4], is seventh highest in the world for the number of mobile subscriptions [3,5].

The mobile phone has shown remarkable promise in different areas of human endeavor, including health care, commerce, aviation, and entertainment [6]. The potential of mobile telephony to enhance health care and improve health research has been recognized by health care practitioners and researchers worldwide [7-10]. Numerous mHealth interventions have been implemented all over the world with varying degrees of success [11-14]. Furthermore, the use of the mobile phone to enhance the conduct of health research in different settings has been explored widely, with mobile phone–based apps being developed for surveys and follow-up of study participants, among others [15,16]. In many of these interventions, the success of mHealth interventions has been generally limited. Reasons for this have included weak surveillance, drug and logistic stockouts, and a lack of skilled human resources [17,18].

The widespread ownership and use of the mobile phone has been touted as one of the strengths of implementing mobile phone–based health interventions [9]. To benefit maximally from this technology, stakeholders have recommended several strategies to entrench mHealth into national health systems—for example, forging strategic partnerships [19], securing an appropriate policy environment [1,20], and stimulating national political commitment and ownership [19].

Nigeria, recognizing the immense potential of information and communication technology in health care, has developed a policy framework to enhance information and communication technology infrastructure to support efforts toward universal health coverage [21]. Among other things, this policy seeks to provide standards for eHealth and mHealth implementation within a proper governance structure. In the light of this work, the mobile phone, which is ubiquitous, will play a key role in furthering these objectives [21].

There is, however, limited evidence of the reliability of self-reported mobile phone ownership and access, which may be affected by various social and infrastructural factors. This limited reliability of self-reported phone ownership may also affect the effectiveness of mobile phone–based interventions in the event that reliability is less than anticipated.

In this study, we sought to ascertain the reliability of self-reported mobile phone ownership and access, in 7 local government areas in Benue State, north-central Nigeria. This information will inform planning and implementation of future mHealth interventions to maximize their impact and reach.

### Preliminary Study: The Healthy Beginning initiative

The Healthy Beginning Initiative (HBI) was a US National Institutes of Health–funded, cluster randomized trial designed to evaluate the comparative effectiveness of church-based, free, confidential, and integrated laboratory testing provided on-site during baby showers for pregnant women and their male partners on HIV testing and linkage to health facilities. This intervention was associated with a higher HIV testing rate (control group: 740/1355, 54.6% vs intervention group: 1514/1647, 91.9%; adjusted odds ratio 11.2, 95% CI 8.77-14.25; *P* ≤.001) [22,23]. HBI used a network of church-based health advisors and clinic-based teams trained in motivational interviewing and quality improvement skills to engage and support HIV-infected women. This program was adopted and scaled up by the US President’s Emergency Plan For AIDS Relief via support to Caritas Nigeria in Benue State to achieve community testing targets.

We drew participants for our study from a database of pregnant women and their male partners who participated in the HBI scale-up effort in Benue State, Nigeria between September 1 and December 31, 2016.

### Aims of the Study

The overarching objective of this study was to determine the reliability of self-reported mobile phone ownership and use as a means of delivering health care interventions. The aims of the study were to determine the proportion of self-reported mobile phone numbers that (1) ring when called, (2) are answered when called, and (3) are answered by the intended participant when called.

## Methods

### Participant Selection and Sample Size Determination

We used a stratified random sampling procedure. We first stratified data by the participants’ sex. Participants were drawn from the HBI program. We selected men and women proportionate to the ratio of female to male participants in the program database (62:38). Sample size for the study was determined by assuming a nominal response rate of 50%, which maximizes the sample-size calculation and a precision of 5%. A total of 2215 participants of known sex in the HBI database had provided telephone numbers. This resulted in a sample size of 328 participants needed for this study. For ease of design and to provide additional participants for robustness, we rounded this number up to 350 participants. Sample-size calculations were performed using the SampSize calculator [24].

### Inclusion and Exclusion Criteria

We included male and female participants more than 18 years of age who participated in HBI in Benue State between September 1 and December 31, 2016, and who reported at least one primary mobile phone number on their biodata collection form. We excluded participants who did not provide a mobile phone number from the dataset before sampling.

**Figure 1:**
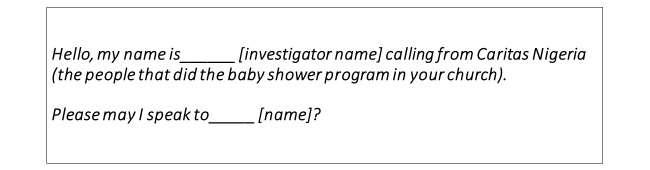
Script used when respondents were called.

### Study Procedures

We developed a script for the phone call to be made to participants. Investigators dialed the participant’s phone number and, if the call was answered, read the script (Figure 1).

If the call was answered by another party, the research assistant asked an additional question to ascertain the relationship between the respondent and the intended participant. If the phone rang, but was not answered, an attempt was made for a follow-up call on the next scheduled day, for up to 3 attempts within a 1-week period. Results of each call were documented on a data collection log. This was entered into a Microsoft Excel 2010 spreadsheet (Microsoft Corporation), and the entries were double-checked by research coordinators to ensure completeness and appropriateness. These data were then deidentified and exported to Stata 13 (StataCorp LLC), which we then used to conduct quantitative analysis.

### Statistical Analyses

We calculated descriptive statistics for selected clinical characteristics. We used the chi-square test to compute the difference in proportion between the different possible outcomes following the call: did not ring; rang but no answer; answered by another party; and answered by the participant. Among the associations we studied were the participant’s sex and marital status. In cases where the calls were answered by individuals other than the intended participants, we drew associations between the participant’s sex and their relationship with the eventual respondent. We analyzed differences in respective proportions using the chi-square test.

## Results

There were a total of 349 participants in this study. Their ages ranged from 18 to 75 years, with a mean age of 27 years. Most respondents were aged between 21 and 30 years (Table 1). There were 216 women and 133 men, in accordance with the ratio of 62:38 (Table 1).

Of the 349 numbers we attempted to call, 123 were not reachable, and 28 rang but there was no answer. For 88 of them, a party other than the participant answered the call. We reached 110 of the participants (31.2%) on the phone (Table 2).

There were significant differences when we stratified by sex the best outcome of the calls made. Among men, we reached 51.1% (68/133) of the participants, compared with 19.4% (42/216) of women (*P*<.001) (Table 3). Among women, 34.3% (n=74) of the calls were answered by another party, while among men, the proportion was 10.5% (n=14). Among women whom we did not reach on the numbers they provided, 54.4% (37/68) of the calls were answered by their husbands and 16.2% (11/68) were marked as wrong numbers. Among men, only 13 of the numbers called were answered by parties other than the intended participants, with only 3 (23.08%) being their spouses (Table 4).

**Table 1:** Participants’ characteristics.

Characteristic		Total (N=349), n (%)
**Age range (years)**		
	≤20	67 (19.2)
	21-30	201 (57.6)
	31-40	68 (19.5)
	41-50	9 (2.6)
	≥51	4 (1.2)
**Sex**		
	Male	133 (38.1)
	Female	216 (61.9)

**Table 2:** Best outcome after a maximum of 3 call attempts.

Best outcome	Total (N=349), n (%)
Did not ring	123 (35.2)
Rang, no answer	28 (8.0)
Rang, answered by another party	88 (25.2)
Rang, answered by participant	110 (31.5)

**Table 3:** Outcome stratified by sex.

Best outcome	Sex, n (%)		Total (N=349), n (%)
	Male (n=133)	Female (n=216)	
Did not ring	44 (33.1)	79 (36.6)	123 (35.2)
Rang, no answer	7 (5.3)	21 (9.7)	28 (8.0)
Rang, answered by another party	14 (10.5)	74 (34.3)	88 (25.2)
Rang, answered by participant^a^	68 (51.1)	42 (19.4)	110 (31.5)

^a^*P*<.001.

**Table 4:** Relationship to participant when the participant’s phone rang but was answered by another party^a^.

Relationship to participant	Sex of study participant, n (%)		Total (N=81), n (%)
	Male (n=13)	Female (n=68)	
Brother	2 (15)	2 (3)	4 (5)
Brother-in-law	0 (0)	6 (9)	6 (7)
Father	2 (15)	0 (0)	2 (2)
Friend	1 (8)	1 (2)	2 (2)
Spouse	37 (54)	3 (23)	40 (49)
Mother	0 (0)	2 (3)	2 (2)
Neighbor	1 (8)	5 (7)	6 (7)
Sister	1 (8)	2 (3)	3 (4)
Sister-in-law	0 (0)	1 (2)	1 (1)
Son	0 (0)	1 (2)	1 (1)
Wrong number	3 (23)	11 (16)	14 (17)

^a^The relationship to the participant was not specified in a few cases.

## Discussion

### Principal Findings

Our study examined the reliability of self-reported mobile phone ownership and access in a predominantly rural area in north-central Nigeria. Our findings show that, in spite of high reported mobile phone ownership among participants, only one-third of these numbers were answered by those who reported ownership of those phones when called.

An unusually high proportion (35.2%) of calls we made did not connect. This may be explained by participants giving incorrect numbers. Incorrect numbers being called may have been a result of the generally low level of education among respondents or data entry errors. Other plausible reasons for calls not successfully connecting are the erratic power supply and poor network connectivity in some areas, which have long been recurring problems in Nigeria [25]. This reduces the reliability of household items, such as the mobile phone, that rely primarily on electricity to function. This may partly account for the unusually high proportion of calls that did not ring. Some of the phones that did not ring may have had dead batteries as a result of prolonged periods of power outage.

Of the calls that were answered by parties other than the intended respondents, 82.7% (67/81) were people who knew the participants, revealing the extent to which mobile phones are shared in households. These findings are consistent with those from a survey conducted in a similar setting in Ghana, which found that the practice of phone sharing was common, especially in rural areas [26].

We also observed that only a small proportion of the calls actually connected but were not answered (8.02%). This may be explained by the fact that participants were informed at enrollment that they would be contacted on their phones and so may have expected our calls, and there is no cost associated with answering phone calls. The population we surveyed was also rural, and most participants were known to not use texts or social media because of low literacy. It is, however, noteworthy that a small proportion of this group may not have answered the calls because they did not recognize the number we used to contact them.

In addition, there were significant differences in control and accessibility of the phones between men and women. This was demonstrated by the huge disparity in the percentage of wives and husbands who answered calls meant for their partners. These findings may be explained by the different gender roles common in many low- and middle-income countries, where men are more economically empowered and women play a subservient role [27]. These traditional gender roles may also explain the difference in outcomes between men and women, where less than half of women were actually reached after 3 attempts.

The results of this study can be more generally applied to other areas within and outside of Nigeria with a similar demographic and socioeconomic structure. A potential limitation of our study may be the nongeneralizability of our findings to more urban populations with higher levels of education and less adherence to traditional gender roles, such as higher-income countries or places within low- and middle-income countries where people do not adhere to traditional gender roles and have higher levels of education.

In light of these findings, certain considerations should be taken into account when designing studies or interventions that depend on participants’ self-reported ownership of or access to mobile phones, especially in rural areas. The less-than-optimal and gendered pattern of actual phone access should inform methodology for research and programs, particularly in low- and middle-income areas. These studies should target larger sample sizes or use alternative means of contacting participants due to the possibility of high nonresponse rates.

### Conclusion

This study demonstrated that reported mobile phone ownership and access, especially in rural and low-income settings, is higher than what exists in reality. In addition, the high proportion of calls that were answered by husbands of participants is noteworthy. This has implications for mHealth programs, for the purposes of either data gathering or the implementation of health interventions, particularly those that target female participants and their health. mHealth practitioners in these areas therefore need to be cognizant of this and adjust appropriately when planning their programs. This lower-than-expected actual access may also explain the mixed results produced by mHealth interventions, particularly those that depend on phones owned by the intended beneficiaries of these programs.

## Abbreviations

HBI: Healthy Beginning initiative
